# Supplementary material for: REST elevation-dependent chromatin remodeling and alternative Grk6 transcript synthesis hyperactivates Cxcr4-Sdf1 signaling in cerebellar granule cell progenitors
Source: bioRxiv. 2025 Oct 15:2025.10.15.682654. Preprint. [Version 1] doi: 10.1101/2025.10.15.682654 (PMC12632871; doi:10.1101/2025.10.15.682654)
Supplement: Supplement 1 [file media-1.pdf]

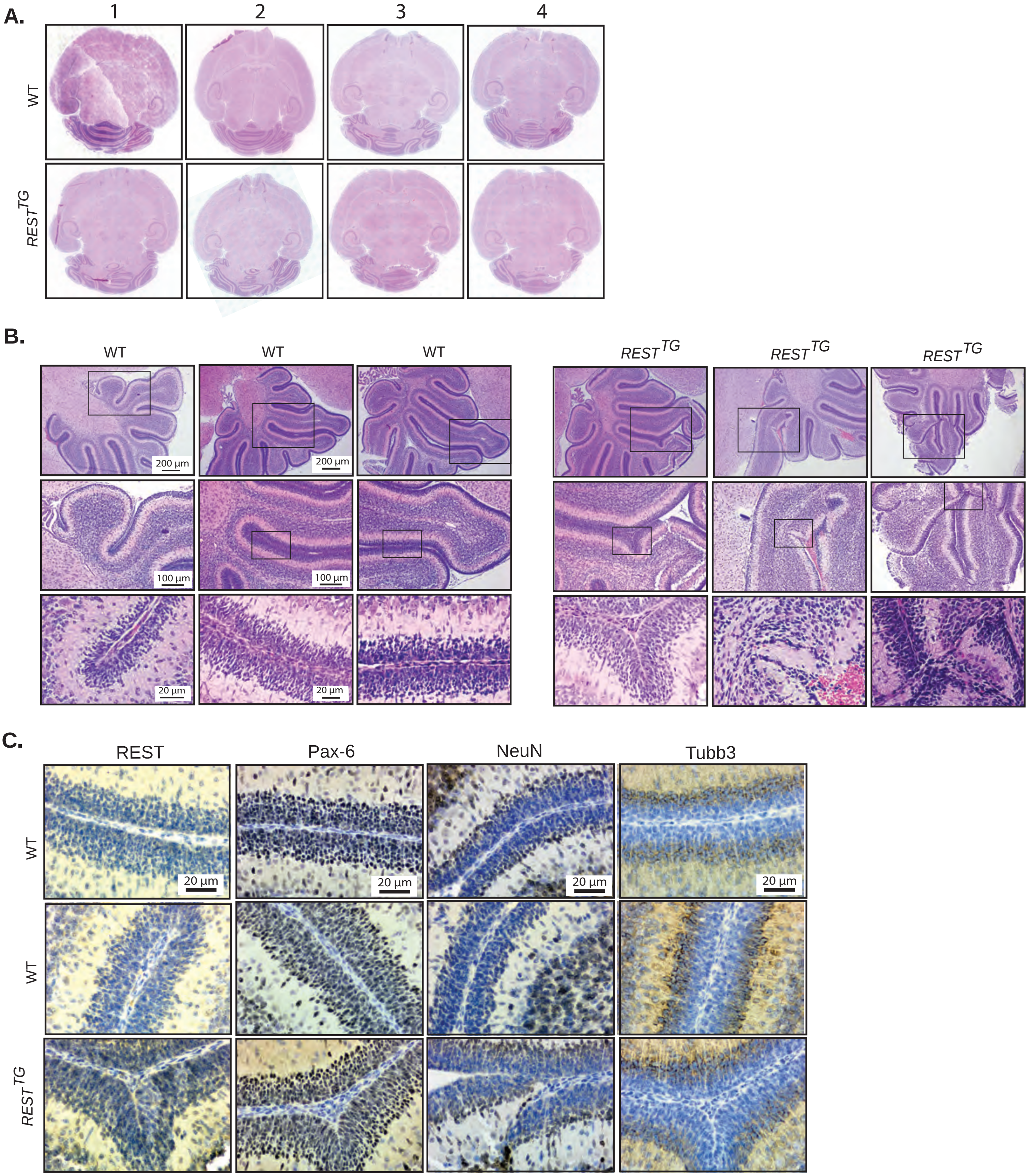
A.
1
2
3
4
WT
REST^TG
B.
WT
WT
WT
REST^TG
REST^TG
REST^TG
200 µm
200 µm
100 µm
100 µm
20 µm
20 µm
C.
REST
Pax-6
NeuN
Tubb3
WT
WT
REST^TG
20 µm
20 µm
20 µm
20 µm

Supp. Fig. 1

D.

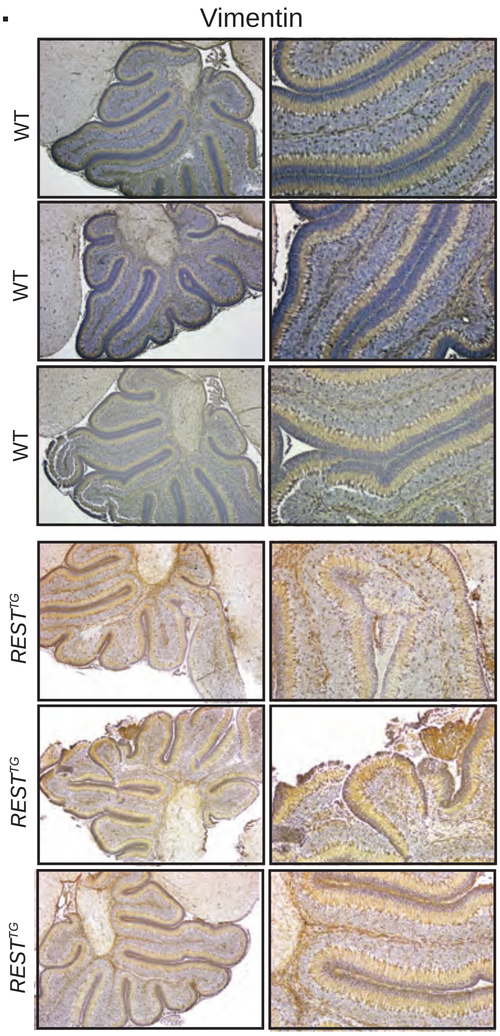

E.

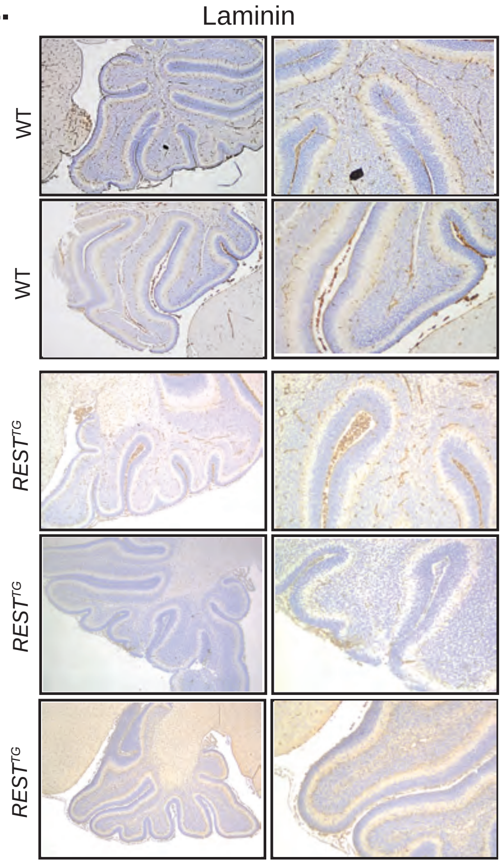

F.

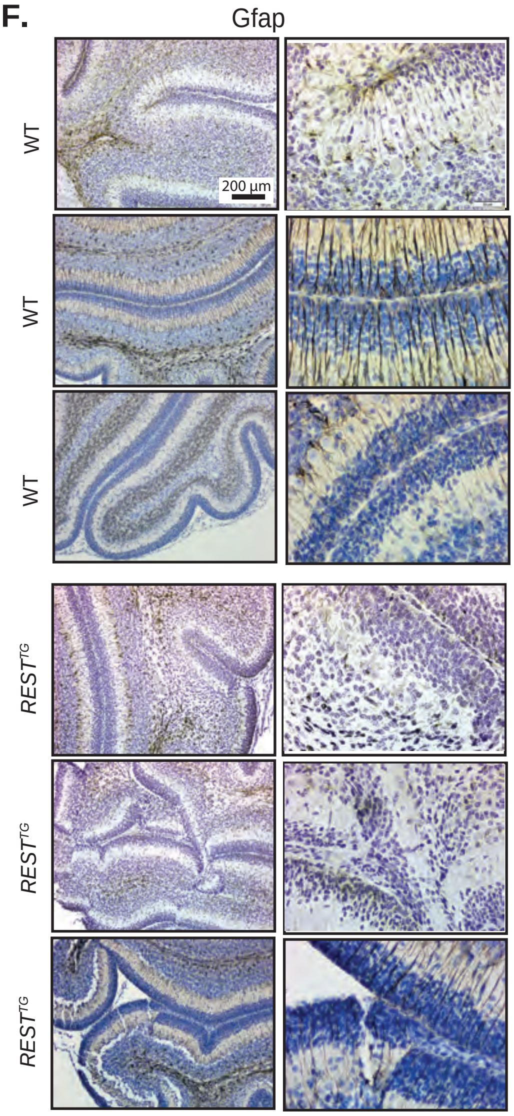

G.

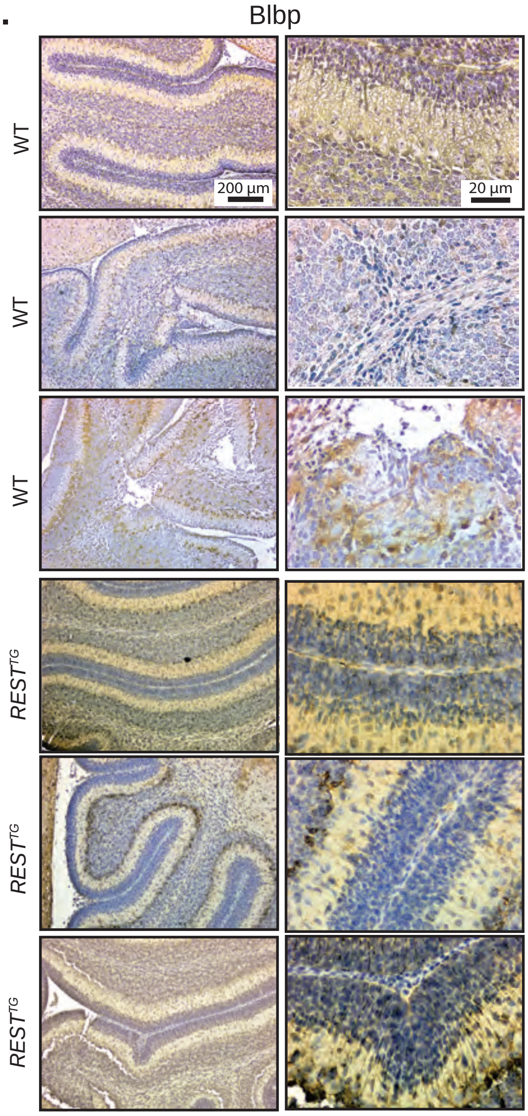

Supp. Fig. 1

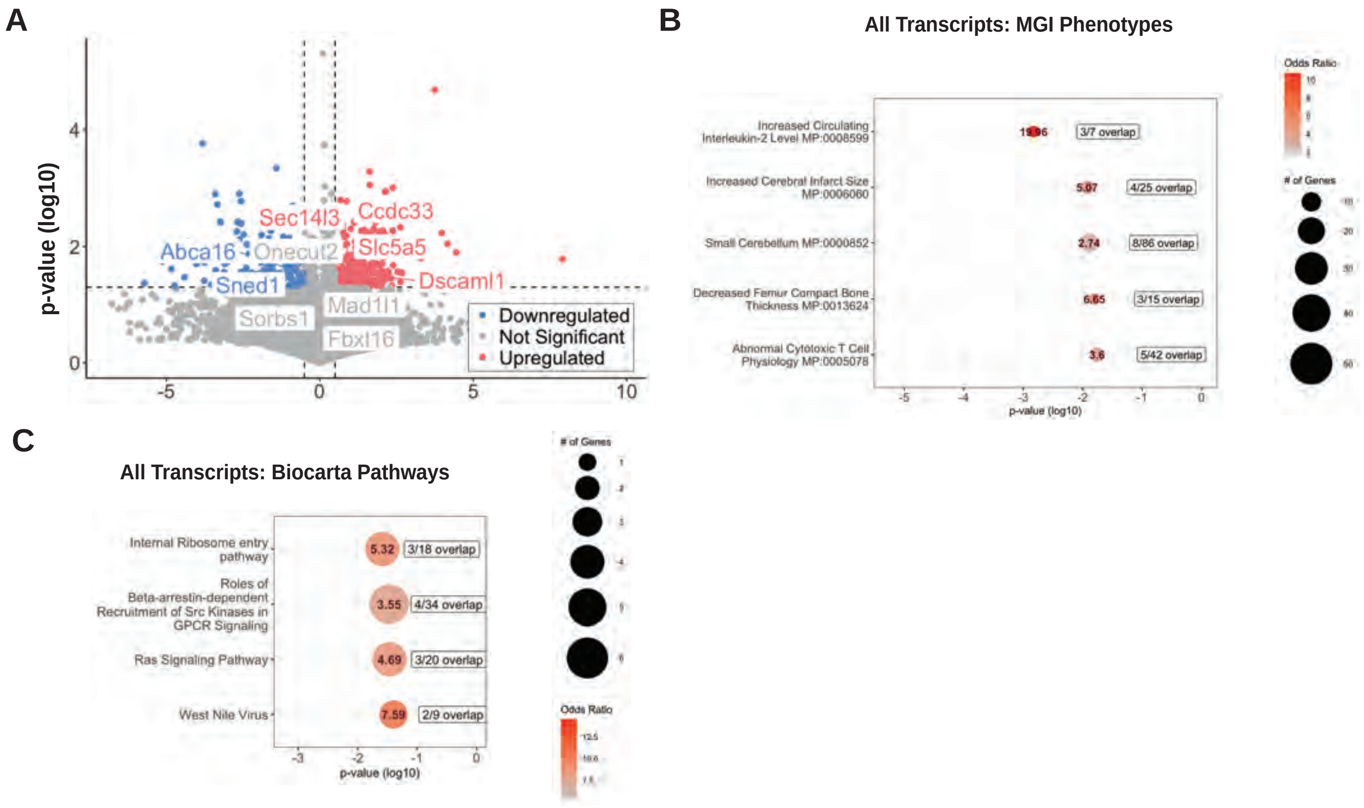
A
p-value (log10)
Sec14l3
Ccdc33
Abca16
Onecut2
Slc5a5
Sned1
Dscaml1
Mad1l1
Sorbs1
Fbxl16
Downregulated
Not Significant
Upregulated
B
All Transcripts: MGI Phenotypes
Increased Circulating Interleukin-2 Level MP:0008599
3/7 overlap
Increased Cerebral Infarct Size MP:0006060
5.07
4/25 overlap
Small Cerebellum MP:0000852
2.74
8/86 overlap
Decreased Femur Compact Bone Thickness MP:0013624
6.65
3/15 overlap
Abnormal Cytotoxic T Cell Physiology MP:0005078
3.6
5/42 overlap
p-value (log10)
Odds Ratio
# of Genes
C
All Transcripts: Biocarta Pathways
Internal Ribosome entry pathway
5.32
3/18 overlap
Roles of Beta-arrestin-dependent Recruitment of Src Kinases in GPCR Signaling
3.55
4/34 overlap
Ras Signaling Pathway
4.69
3/20 overlap
West Nile Virus
7.59
2/9 overlap
p-value (log10)
# of Genes
Odds Ratio

Supp. Fig. 2

Supplemental Figure 2A. RNA-sequencing pathway analysis.

Genes with putative RE-1 motifs ($p<0.05$)

No.	Gene names	RNAseq FC	No.	Gene names	RNAseq FC
1	Abhd3	-0.6995336	43	Pex5l	-0.6319134
2	Actg2	1.41578462	44	Pla2g7	-1.2099169
3	Adam18	-1.1801238	45	Plxnb1	-0.5217478
4	Adgrb1	-0.580416	46	Psg27	1.12132482
5	AI467606	0.75599525	47	Rgl1	0.53987986
6	Aldh3a1	-2.2369835	48	Rims3	0.58440633
7	Arhgap22	-1.1576724	49	Rtn4rl2	0.77305452
8	Asic1	0.65879247	50	Scrt1	-0.8519393
9	Bmp7	0.53336413	51	Selenbp1	0.96091995
10	Btc	-1.3249227	52	Serpinb6d	1.08920012
11	Cabp5	-0.7418395	53	Sh3rf3	0.56187138
12	Car12	-0.8338592	54	Slc22a18	-1.1387966
13	Casp8	-0.5014259	55	Slc43a2	-0.9742032
14	Ccdc148	-0.9121784	56	Slc8a1	-0.5959781
15	Ceacam19	1.72496176	57	Slco6d1	-1.4307103
16	Col8a1	-0.9984369	58	Spaca3	-1.3905089
17	D630003M2Rlk	-0.719174	59	Spats2l	0.75242876
18	Efna2	0.51038	60	Spdl1	0.51862748
19	Ehbp1l1	-1.1900377	61	Srd5a2	-1.1834461
20	Eln	0.57239964	62	Taar8a	-2.2733348
21	Enkur	-0.8510465	63	Tnfsf14	1.08443682
22	Fcgbp	0.6683151	64	Tppp2	1.74020861
23	Fev	1.69746166	65	Trabd2b	-1.0176281
24	Fgf11	0.59104378	66	Trim30c	-2.1764693
25	Fgf18	0.81632677	67	Trpm8	0.69151487
26	Frmd6	0.52545867	68	Unc93a2	0.56692521
27	Galnt6	-1.0587295	69	Vav1	-0.71402
28	Gem	-1.0587295	70	Vmn2r107	-2.0006126
29	Hnf1a	0.83678178	71	Wnt7b	-0.5071865
30	Hsf5	0.65288483	72	Zdhhc11	1.0196227
31	Ireb2	-0.619702			
32	Islr	-1.0817079			
33	Itgb4	0.56790881			
34	Mov10l1	0.75920618			
35	Mrap2	-0.7363836			
36	Mrgpra1	-2.7067668			
37	Mycbpap	0.97284589			
38	Myo16	-0.5179541			
39	Nmur1	-1.1027255			
40	Nt5c1b	1.29378181			
41	Oc90	1.04724261			
42	Pde6b	0.69100674			

Supplemental Figure 2B. RNA-sequencing pathway analysis.

No.	Genes with putative RE-1 motifs (p<0.05) & significant DE (p<0.05)	RNAseq FC
1	Actg2	1.41578462
2	Adam18	-1.1801238
3	Aldh3a1	-2.2369835
4	Arhgap22	-1.1576724
5	Btc	-1.3249227
6	Ceacam19	1.72496176
7	Ehbp1l1	-1.1900377
8	Fev	1.69746166
9	Galnt6	-1.0587295
10	Islr	-1.0817079
11	Mrgpra1	-2.7067668
12	Nmur1	-1.1027255
13	Nt5c1b	1.29378181
14	Oc90	1.04724261
15	Pla2g7	-1.2099169
16	Psg27	1.12132482
17	Serpinb6d	1.08920012
18	Slc22a18	-1.1387966
19	Slco6d1	-1.4307103
20	Spaca3	-1.3905089
21	Srd5a2	-1.1834461
22	Taar8a	-2.2733348
23	Tnfsf14	1.08443682
24	Tppp2	1.74020861
25	Trabd2b	-1.0176281
26	Trim30c	-2.1764693
27	Vmn2r107	-2.0006126
28	Zdhhc11	1.0196227

A

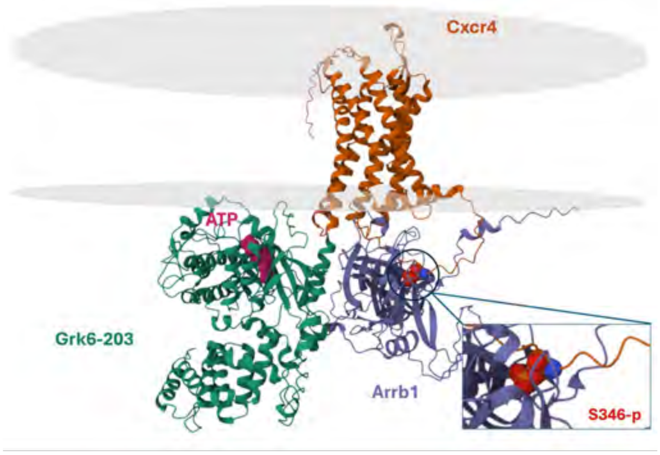

B

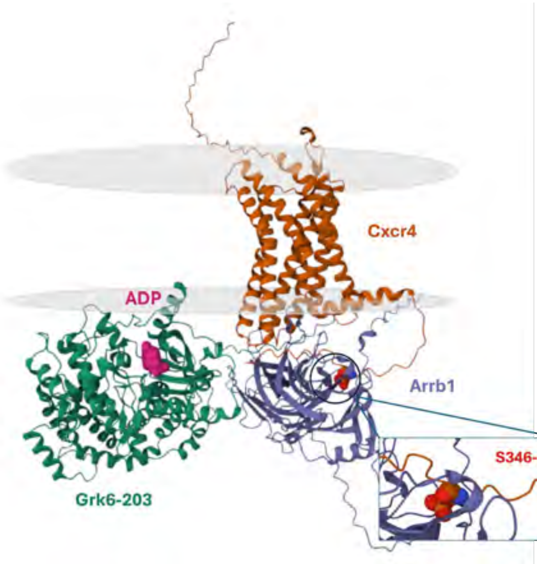

C

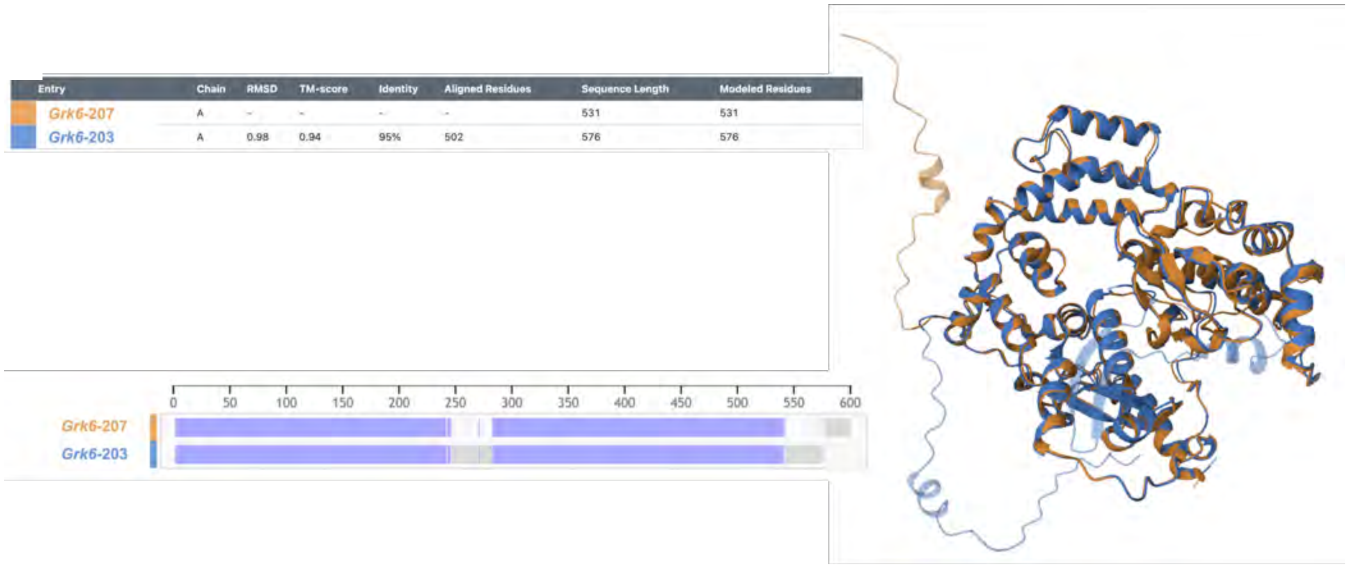

Entry	Chain	RMSD	TM-score	Identity	Aligned Residues	Sequence Length	Modeled Residues
Grk6-207	A	-	-	-	-	531	531
Grk6-203	A	0.98	0.94	95%	502	576	576

D

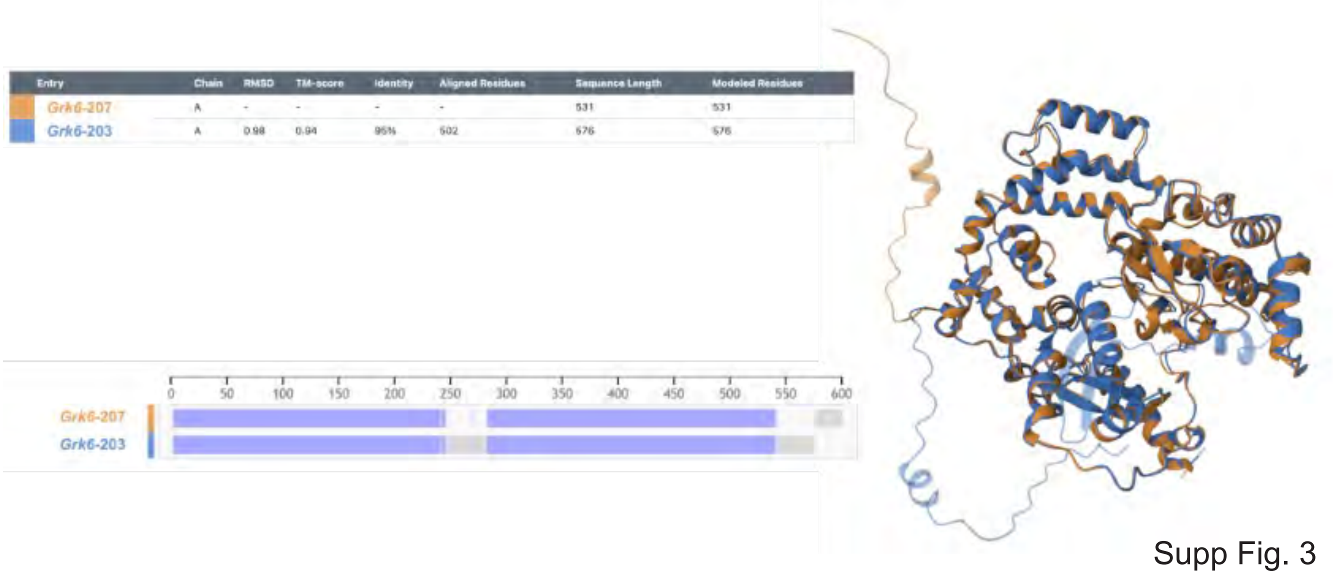

Entry	Chain	RMSD	TM-score	Identity	Aligned Residues	Sequence Length	Modeled Residues
Grk6-207	A	-	-	-	-	531	531
Grk6-203	A	0.98	0.94	95%	502	576	576

Supp Fig. 3

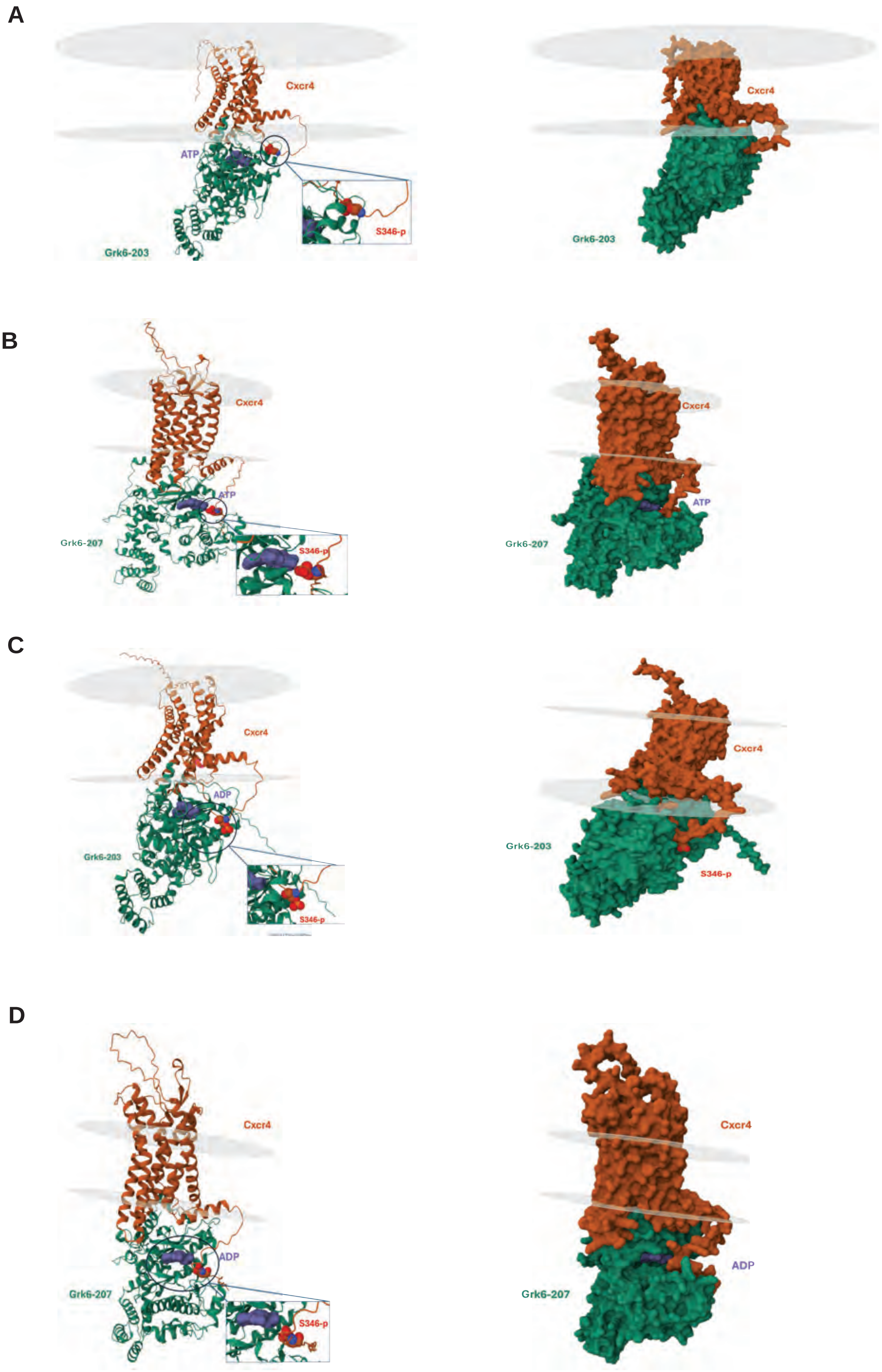

A
Cxcr4
ATP
S346-p
Grk6-203
Cxcr4
Grk6-203
B
Cxcr4
ATP
Grk6-207
S346-p
Cxcr4
ATP
Grk6-207
C
Cxcr4
ADP
Grk6-203
S346-p
Cxcr4
Grk6-203
S346-p
D
Cxcr4
ADP
Grk6-207
S346-p
Cxcr4
ADP
Grk6-207

Supp. Fig. 4

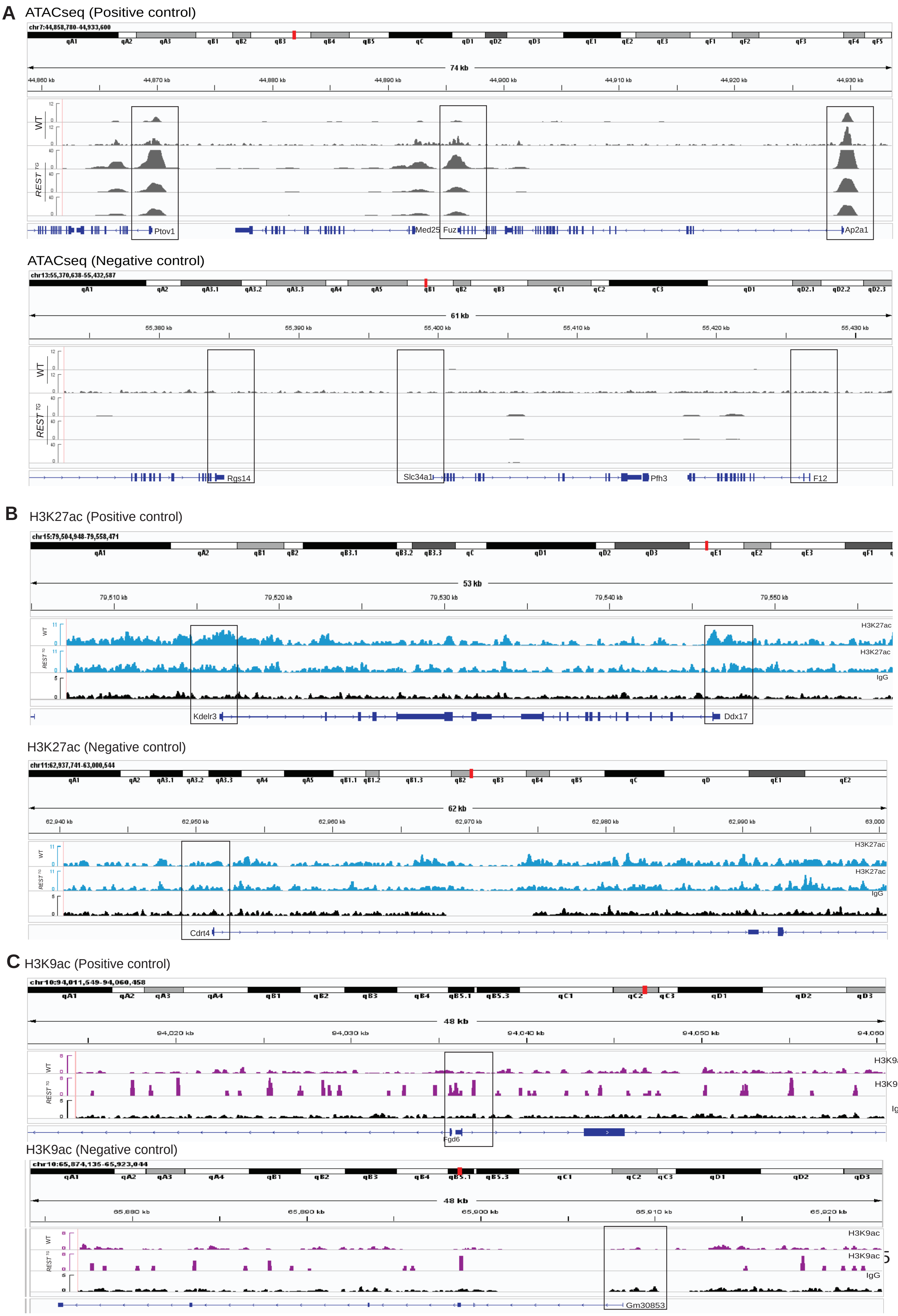
A
ATACseq (Positive control)
chr7:44,858,780-44,933,600
74 kb
WT
REST TG
Ptov1
Med25
Fuz
Ap2a1
ATACseq (Negative control)
chr13:55,370,638-55,432,587
61 kb
WT
REST TG
Rgs14
Slc34a1
Pfh3
F12
B
H3K27ac (Positive control)
chr15:79,504,948-79,558,471
53 kb
WT
REST TG
H3K27ac
H3K27ac
IgG
Kdelr3
Ddx17
H3K27ac (Negative control)
chr11:62,937,741-63,000,544
62 kb
WT
REST TG
H3K27ac
H3K27ac
IgG
Cdrt4
C
H3K9ac (Positive control)
chr10:94,011,549-94,060,458
48 kb
WT
REST TG
H3K9ac
H3K9ac
IgG
Fgd6
H3K9ac (Negative control)
chr10:65,874,135-65,923,044
48 kb
WT
REST TG
H3K9ac
H3K9ac
IgG
Gm30853

D H3K4me3 (Positive control)

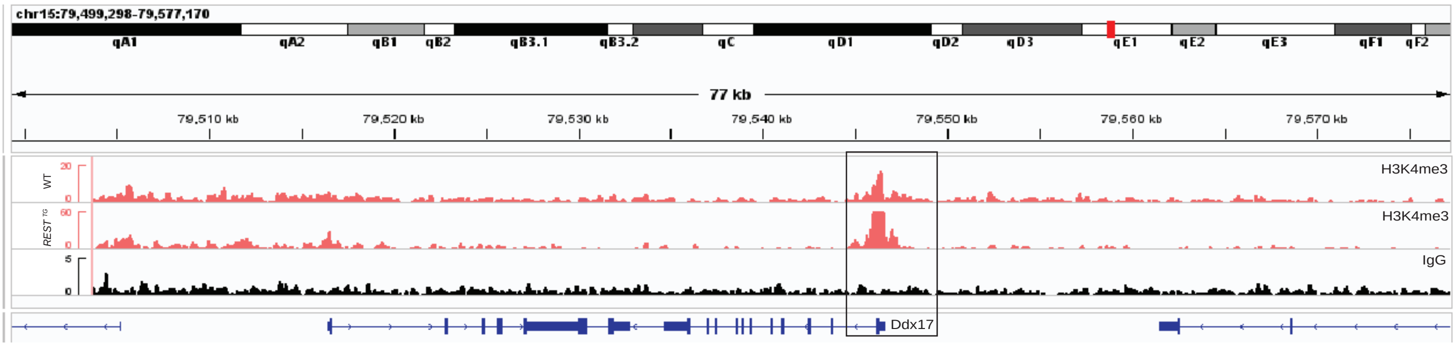

H3K4me3 (Negative control)

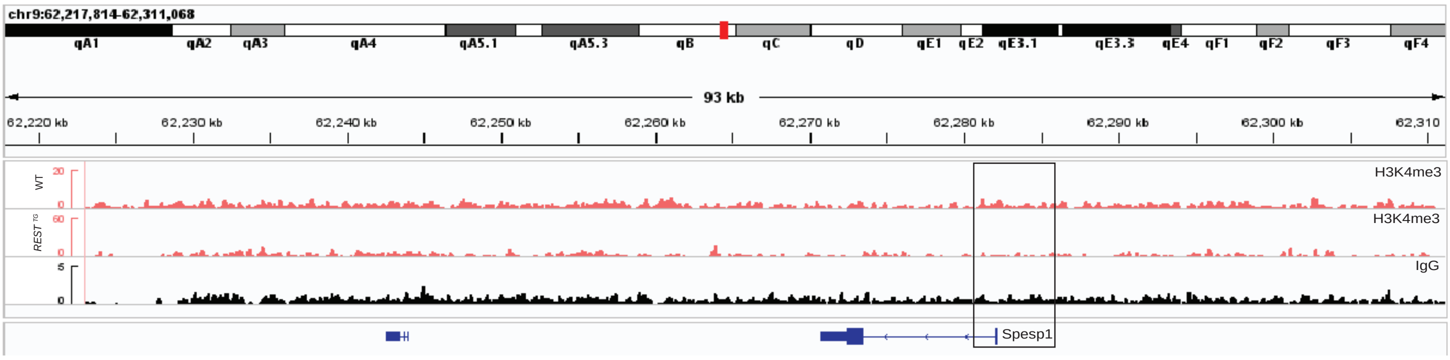

E Ezh2 ChIPseq target genes (mouse)

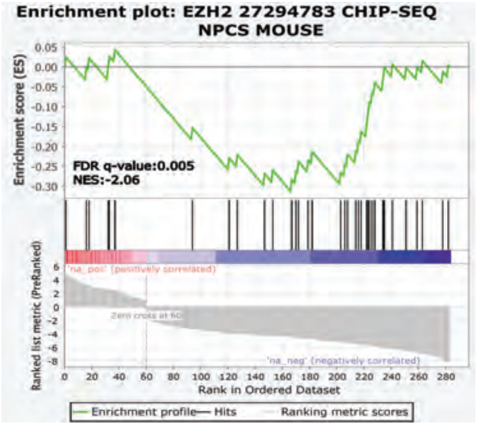

A Ezh2 and H3K27me3 (Positive control)

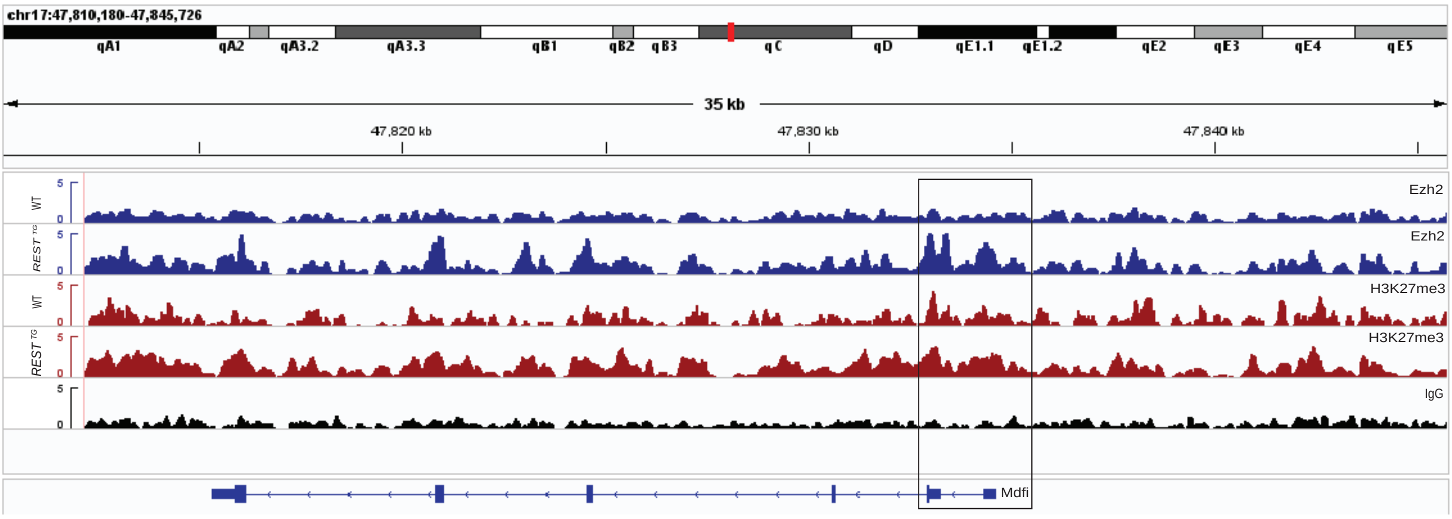

Ezh2 and H3K27me3 (Negative control)

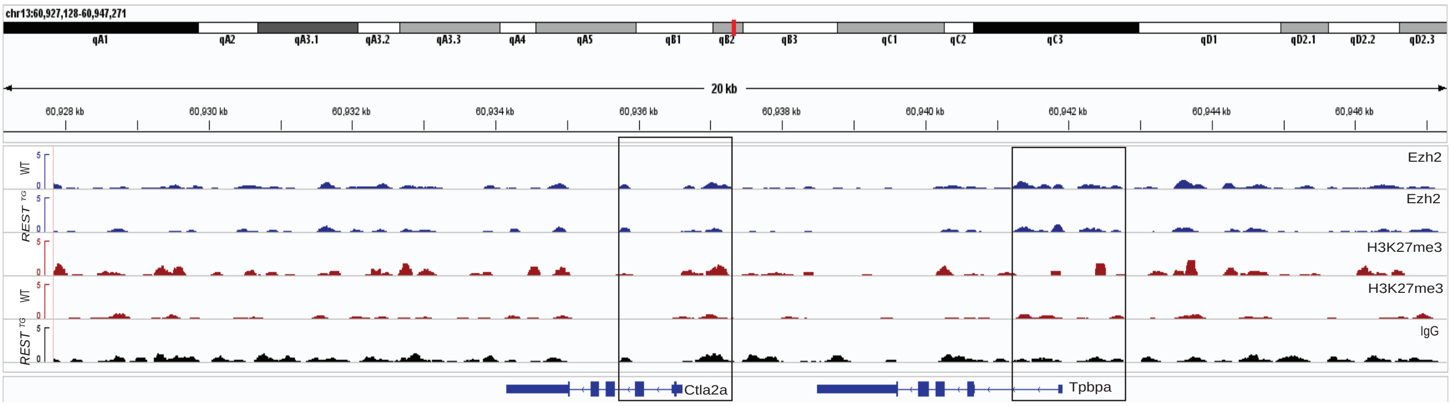

B H3K36me3 (Positive control)

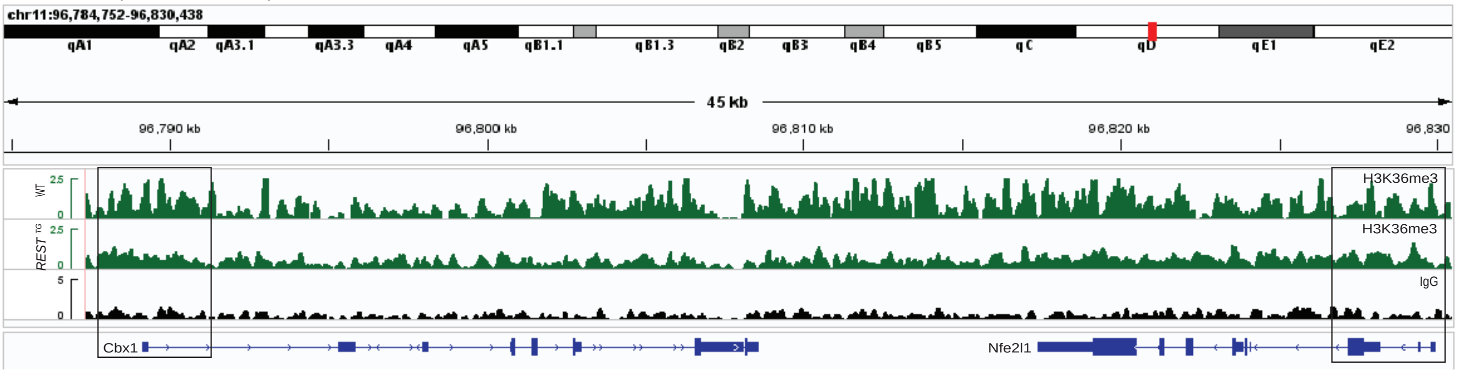

H3K36me3 (Negative control)

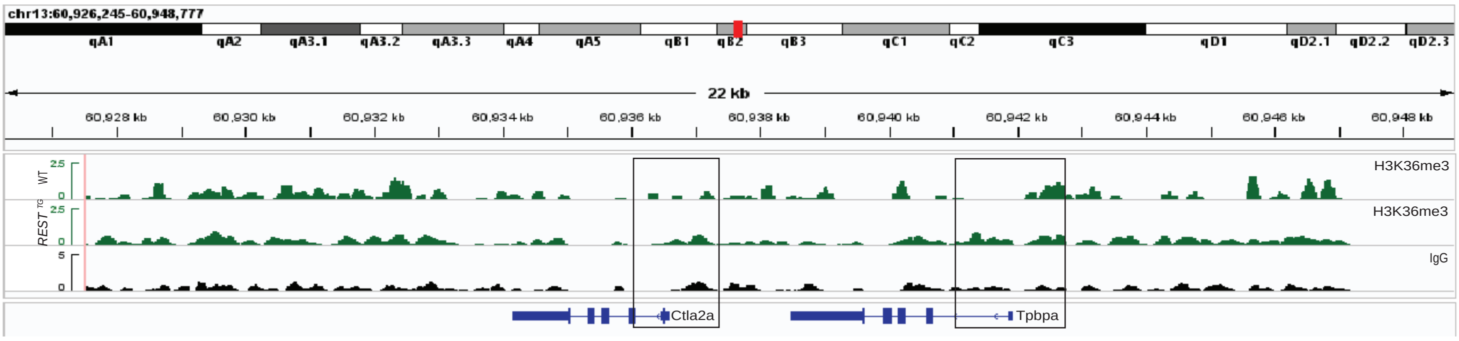

Supp. Fig. 6

Supplementary figure legends:

Figure S1. REST overexpression disrupts normal cytoarchitecture in the postnatal cerebellum. **(A)** H&E of additional samples of horizontal brain slices from Wildtype (WT) and *REST*^{TG} mice used for symmetry analysis. **(B)** H&E staining of sagittal sections of cerebella from WT and *REST*^{TG} mice in two additional *REST*^{TG} lineages. Boxes in upper panels correspond to the frame of the next row. Scale bars in left panels apply to all panels within row. IHC for **(C)** REST, Pax6, NeuN, Tubb3, **(D)** Vimentin, **(E)** Laminin **(F)** Gfap, and **(E)** Blbp in sagittal sections of the cerebella of additional WT and *REST*^{TG} mice. Scale bars in top panels apply to all panels within column.

Figure S2. Differential gene expression and pathway analysis of RNAseq data of wildtype and *REST*^{TG} CGNPs.

(A) Volcano plot of gene expression between WT and *REST*^{TG} cGNPs with highlighted genes representing those with significant differential expression $\geq \pm 1$ Log2FC. **(B)** Top 5 enriched and nominally significant ($p<0.05$) mouse genome informatics (MGI) phenotypes (left) and **(C)** all significantly enriched molecular pathways (Biocarta; right) for all nominally significant genes with differential expression ($p<0.05$.

Figure S3. Comparison of Alphafold3 stick/ribbon structural predictions for Grk6-203 and Grk6-207.

Stick/ribbon structures of the Alphafold3 structural predictions using amino acid sequences of Grk6-203 (WT) and canonical mouse Cxcr4 and Arrb1 in the presence of **(A)** ATP and **(B)** ADP. Grey discs represent borders of the plasma membrane bilayer. Surface representations of predictions are provided in upper border boxes. Zoomed view of the phosphorylated S346 is provided in bottom bordered boxes. **(C) and (D)** Variation in primary sequences of Grk6-203 and Grk6-207 are shown. Adjacent ribbon/stick figures are superimposed structures of Grk6-203 and Grk6-207 showing the differences.

Figure S4. Comparison of Alphafold3 stick/ribbon structural predictions for Grk6-203 and Grk6-207 interaction with Cxcr4 in the presence ATP or ADP.

Stick/ribbon structures and surface plots of the Alphafold3 structural predictions using amino acid sequences of Grk6-202 (WT) and canonical mouse Cxcr4 in the presence of ATP **(A)** and **(C)** and in the presence of ADP **(B)** and **(D)** ADP. Grey discs represent borders of the plasma membrane bilayer. Surface representations of predictions are provided in upper border boxes. Zoomed view of the phosphorylated S346 is provided in bottom bordered boxes.

Figure S5. Positive and negative controls for ATACseq and ChIPseq analyses in WT and *REST^TG* CGNPs.

(A) Differences in chromatin accessibility at positive (*Ptov1, Fuz* and *Ap2a1*) and negative (*Rgs14 and Slc34a*) control gene loci in WT and *REST^TG* CGNPs was measured by ATACseq. **(B)** Enrichment of H3K27ac at positive (*Kdelr3* and *Ddx17*) and absence of enrichment in negative controls genes (*Cdrl4*) are shown. **(C)** Enrichment of H3K9ac at positive (*Fgd6*) and its absence in negative controls genes (*Gm30853*) are shown. **(D)** Enrichment of H3K4me3 at positive (*Ddx17*) and its absence in negative controls genes (*Spesp1*) are shown. **(E)** GSEA analysis to identify enrichment of Ezh2 ChIPseq target genes among 427 coordinated genes.

Figure S6. Positive and negative controls for ChIPseq analysis for EZH2 occupancy and enrichment of H3K27me3 and H3K36me3 in WT and *REST^TG* CGNPs.

(A) Enrichment of Ezh2 and H3K27me3 at positive (*Mdfi*) and its absence in negative controls genes (*Ctla2a* and *Tpbpa*) are shown. **(B)** Enrichment of H3K36me3 at positive (*Cbx1* and *Nfe2l1*) and absence of enrichment in negative controls genes (*Ctla2a* and *Tpbpa*) are shown.

Table S1

Antibodies - IHC

**Antibody**	**Catalog Number**	**Dilution**	**Company**
REST	00141	1:50	Bethyl Laboratories, Montgomery TX, USA
Vimentin	ab92547	1:200	Abcam, Cambridge, UK
Laminin	ab11575	1:200	Abcam, Cambridge, UK
Tubb3	801201	1:250	Biolegend, San Diego CA, USA
NeuN	ab13938	1:200	Abcam, , Cambridge, UK
Gfap	3670	1:50	Cell Signaling Technology, Danvers, MA, USA
Sdf1	MAB350	1:100	R&D Systems, Minneapolis MN, USA
Pax-6	PRB-278P	1:200	Biolegend, San Diego CA, USA
Blbp	ab32423	1:200	Abcam, Cambridge, UK

Table S2

Antibodies - Western

**Antibody**	**Catalog number**	**Dilution**	**Company**
REST	07-579	1:1000	Millipore, Burlington, MA, USA
ERK1/2	9102	1:1000	Cell Signaling Technology, Danvers, MA, USA
p-Erk	9101	1:5000	Cell Signaling Technology, Danvers, MA, USA

p-CXCR4	59028S	1:1000	Cell Signaling Technology, Danvers, MA, USA
p-CXCR4	ab74012	1:1000	Abcam, Cambridge, UK
CXCR4	ab124824	1:2000	Abcam, Cambridge, UK
Sdf1	MAB350	1:500	R&D Systems, Minneapolis MN, USA
Vimentin	ab92547	1:1000	Abcam, Cambridge, UK
Actin	ab7810	1:50000	Abcam, Cambridge, UK

Table S3

qRT-PCR primers mouse CGNPs (WT vs REST^{TG})

**Primer Name**	**Primer Sequence**
mGrk6 IG For	CACCTTCAGGCAGTACCGAG
mGrk6 IG Rev	CACAGCAGATCTCAGCAGCA
m18S F1617	GCAATTATTCCCCATGAACG
m18S R1739	GGCCTCACTAAACCATCCAA
mGrk6 For 1	TGAGATGGTGGAGACCGAGT
mGrk6 Rev 1	CTGCAGTTCCCACAGCAATC
mGrk6 For 2	TTTGCCGACTACCTCGACAG
mGrk6 Rev 2	GTACATCTTGCCTGTTGCCC

Table S4

Antibodies - ChIP

Antibody	Catalog No.	Dilution	Company
H3K9Ac	9649	1:200	Cell Signaling Technology, Danvers, MA, USA
H3K27me3	9733	1:200	Cell Signaling Technology, Danvers, MA, USA
H3K36me3	4909	1:200	Cell Signaling Technology, Danvers, MA, USA
H3K27ac	8173	1:200	Cell Signaling Technology, Danvers, MA, USA
Ezh2	5246	1:200	Cell Signaling Technology, Danvers, MA, USA

Table S5

qPCR primers - ChIP

Primer name	Primer Sequence
mGrk6 TSS1-F	CAGAGGCTCGGCCCTAATTG
mGrk6 TSS1-R	CGATTTCTGGTTGGACTGCG
RE1 mGrk6 F	ACGCATTGTGTACAGGTAGGG
RE1 mGrk6 R	GAGCTGCCACTCTCAAACCT
